# Early outcomes of moderate-to-high-risk pediatric congenital cardiac surgery and predictors of extracorporeal circulatory life support requirement

**DOI:** 10.3389/fped.2024.1282275

**Published:** 2024-03-08

**Authors:** Mimi Xiaoming Deng, Christoph Haller, Kasey Moss, Sudipta Saha, Kyle Runeckles, Chun-Po Steve Fan, Bhavikkumar Langanecha, Alejandro Floh, Anne-Marie Guerguerian, Osami Honjo

**Affiliations:** ^1^ Division of Cardiovascular Surgery, The Labatt Family Heart Centre, The Hospital for Sick Children, Toronto, ON, Canada; ^2^ Department of Surgery, University of Toronto, Toronto, ON, Canada; ^3^ Faculty of Internal Medicine, McMaster University, Hamilton, ON, Canada; ^4^ Rogers Computational Program, Peter Munk Cardiac Centre, University Health Network, Toronto, ON, Canada; ^5^ Department of Critical Care Medicine, Labatt Family Heart Centre, Toronto, ON, Canada

**Keywords:** extracorporeal circulatory membrane oxygenation, congenital, surgery, cardiac, heart, intensive care

## Abstract

**Background:**

Cardiopulmonary failure refractory to medical management after moderate-to-high-risk congenital cardiac surgery may necessitate mechanical support with veno-arterial extracorporeal membrane oxygenation (ECMO). On the extreme, ECMO can also be initiated in the setting of cardiac arrest (extracorporeal cardiopulmonary resuscitation, ECPR) unresponsive to conventional resuscitative measures.

**Methods:**

This was a single-center retrospective cohort study of patients (*n* = 510) aged <3 years old who underwent cardiac surgery with cardiopulmonary bypass with a RACHS-1 score ≥3 between 2011 and 2014. Perioperative factors were reviewed to identify predictors of ECMO initiation and mortality in the operating room (OR) and the intensive care unit (ICU).

**Results:**

A total of 510 patients with a mean surgical age of 10.0 ± 13.4 months were included. Among them, 21 (4%) patients received postoperative ECMO—12 were initiated in the OR and 9 in the ICU. ECMO cannulation was associated with cardiopulmonary bypass duration, aortopulmonary shunt, residual severe mitral regurgitation, vaso-inotropic score, and postprocedural lactate (*p* < 0.001). Of the 32 (6%) total deaths, 7 (22%) were ECMO patients—4 were elective OR cannulations and 3 were ICU ECPR. Prematurity [hazard ratio (HR): 2.61, *p* < 0.01), Norwood or Damus–Kaye–Stansel procedure (HR: 4.29, *p* < 0.001), postoperative left ventricular dysfunction (HR: 5.10, *p* = 0.01), residual severe tricuspid regurgitation (HR: 6.06, *p* < 0.001), and postoperative ECMO (ECPR: HR: 15.42, *p* < 0.001 vs. elective: HR: 5.26, *p* = 0.01) were associated with mortality. The two patients who were electively cannulated in the ICU survived.

**Discussion:**

Although uncommon, postoperative ECMO in children after congenital cardiac surgery is associated with high mortality, especially in cases of ECPR. Patients with long cardiopulmonary bypass time, residual cardiac lesions, or increased vaso-inotropic requirement are at higher risk of receiving ECMO. Pre-emptive or early ECMO initiation before deterioration into cardiac arrest may improve survival.

## Introduction

In surgical repair of moderate- or high-risk congenital heart disease (CHD), extracorporeal membrane oxygenation (ECMO) is utilized for patients who fail to wean from cardiopulmonary bypass (CPB) and experience intractable low cardiac output syndrome (LCOS), pulmonary hypertension, or sudden cardiac arrest (1, 2). The in-hospital survival of pediatric patients receiving postcardiotomy ECMO ranges from 22% to 49% (3), with some studies reporting worse outcomes in those acutely cannulated during extracorporeal cardiopulmonary resuscitation (ECPR) (4, 5). Our institution's data of 180 consecutive postoperative cardiac surgery ECMO cases from 1990 to 2007 identified neurological complications, renal dysfunction, prolonged ECMO duration greater than 3 days, repeat ECMO initiation, and the lack of a heart transplant exit strategy to be associated with in-hospital mortality (6). Interestingly, the indications for ECMO and ECPR were not associated with mortality in this analysis (6). Ischemic brain injury was the most common cause of death among patients who received ECPR (6). In recent years, surgical and intensive care practices have evolved, making it worthwhile to re-evaluate prognosticators of postoperative ECMO and mortality.

In this report, we summarized our institution's more recent experiences with pediatric patients after moderate-to-high-risk congenital cardiac surgery to identify the predictors of ECMO initiation and its effect on in-hospital and 1-year survival. We anticipated that ECPR is associated with poorer survival than elective ECMO. Being able to recognize the demographic, operative, echocardiographic, and biochemical predictors for ECMO could allow for early ECMO initiation and subsequently improved survival.

## Patients and methods

A total of 510 consecutive children aged under 3 years who underwent surgery with CPB and RACHS-1 score ≤3 at the Hospital of Sick Children in Toronto between September 2011 and November 2014 were identified and retrospectively reviewed. The study was approved by the Research Ethics Board at the Hospital for Sick Children, and patient consent was waived.

### ECMO protocol

All neonates and infants were treated according to a standardized protocol for ECMO as previously described (6, 7). Indications for ECMO included the inability to separate from CPB, LCOS, cardiac arrest (institutional practice of ECPR started in 2000), and shunt thrombosis, with the decision ultimately left to the discretion of the interdisciplinary clinical team. In short, in patients within 1 week following cardiotomy, the aorta and atria were directly cannulated through an open chest for expeditious commencement of support and facilitation of effective open cardiopulmonary resuscitation as required. In older patients and those who received delayed postoperative ECMO, peripheral cannulation via the neck or femoral vessels was performed. In this study, patients were <3 years of age with body weight <20 kg; hence, neck access was used exclusively in cases involving peripheral cannulation. Left heart decompression, for left chamber dilation on echocardiography or significant pulmonary edema on chest radiography, was achieved through left atrial cannulation and drainage, or through surgical or balloon atrial septostomy (8). Pump flow was titrated between 100 and 120 ml/kg/min (150–200 ml/kg/min in aortopulmonary shunt lesion), targeting adequate end-organ perfusion and achieving normalization of arterial blood gases, systemic venous saturation, and lactic acid clearance. Anticoagulation was maintained by a continuous heparin infusion at a rate of 50 U/kg, targeting an activated clotting time of 180–200 s. Broad-spectrum prophylactic antibiotics were routinely given in cases of open chests. Echocardiography was performed frequently to monitor cardiac recovery, left ventricular (LV) thrombus formation, and residual cardiac lesions and to inform discussion for a durable ventricular assist device.

### Data collection

Patient demographics including sex, age, prematurity, birth weight, presence of genetic syndromes, baseline biventricular function, and congenital cardiac anatomy were collected. Operative details including CPB time, aortic cross-clamp time, deep hypothermic circulatory arrest time, and type of procedure were obtained. Ventricular function, residual valvular lesions, and other defects on early postoperative echocardiography were examined. Blood arterial lactate level, pH, oxygen saturation of arterial blood (SaO_2_), vasoactive-inotropic score (VIS), and near-infrared spectroscopy for cerebral oximetry (NIRS) at the time of postrepair transesophageal echocardiography and on admission to intensive care unit (ICU) were documented. The setting of veno-arterial ECMO initiation [i.e., operating room (OR) or ICU] was also noted. For terminology in this study, ECMO encompassed both entities of ECPR and elective ECMO. ECPR was defined on the basis of the Extracorporeal Life Support Organization definition of circulatory support in the context of failure to achieve sustained return of spontaneous circulation using conventional cardiopulmonary resuscitation (CPR) (9). Elective ECMO was defined by mechanical circulatory support for failure to wean from CPB or inadequate support despite maximal inotropic agents. LCOS was defined as a cardiac index <2.2 L/min/m^2^ with signs of end-organ malperfusion in the absence of hypovolemia. Other variables such as delayed chest closure, length of intubation, ICU stay, hospital stay, and death were also analyzed. The primary outcome was in-hospital survival and predictors for ECMO initiation in intraoperative and ICU settings. The secondary outcomes were predictors of postoperative mortality in patients supported with ECMO, compared to those not requiring ECMO support.

### Statistical analysis

Continuous variables were described using mean and standard deviation; categorical variables were described using frequencies and proportions. Between-group (ECMO vs. no ECMO) differences in the continuous and categorical variables were evaluated using the Wilcoxon rank-sum test and Fisher's exact test, respectively. Survival was characterized using the Kaplan–Meier method, and between-group differences were assessed using the log-rank test. The impact of receiving ECMO in ICU among those who did not receive ECMO in the OR was quantified using a multistate model, with “no ECMO in OR” being the initial state, “ECMO in ICU” being the transient state, and “death” being the terminal state. ECMO in OR is considered as a binary outcome, whereas ECMO in ICU and mortality are considered as time-to-event outcomes. Administrative censoring was applied at year 1 for the endpoint of death. Univariate analysis was performed because multivariate analysis was not feasible given the low incidence of ECMO events. All analyses were conducted assuming a significance level of 5% and implemented using R v4.0.3 (10).

## Results

### Patients and operative characteristics

A total of 510 consecutive children were included, of which 21 received postoperative ECMO (Table 1) between postoperative days 0 and 6, with 14 patients cannulated on the day of surgery. The average age of operation in the ECMO group was 4.2 ± 7.8 months, compared to 10.0 ± 13.6 months in the non-ECMO group (*p *= 0.047), with no differences in the prevalence of prematurity. Aortic cross-clamp (140 ± 90 vs. 91 ± 55 min, *p *= 0.03) and CPB duration (250 ± 99 vs. 128 ± 58 min, *p *< 0.001) were significantly longer in the ECMO group. Aortopulmonary shunt, pulmonary arterioplasty, and coronary repair were more prevalent in the ECMO group. On average, patients had normal biventricular function at baseline in both groups.

**Table 1:** Baseline and operative characteristics.

	*n*	Total	*n*	ECMO	*n*	No ECMO	*p*-value
Age at the time of operation (days)	510	301 ± 403	21	192 ± 234	489	305 ± 408	**0**.**047**
Prematurity	413	92 (22.3)	17	4 (23.5)	396	88 (22.2)	1.00
Planned procedure	510	493 (96.7)	21	19 (90.5)	489	474 (96.9)	0.15
CPB duration (min)	505	133 ± 65	20	250 ± 99	485	128 ± 58	**<0**.**001**
Cross-clamp duration (min)	505	93 ± 57	20	140 ± 90	485	91 ± 55	**0**.**03**
Preoperative LV function^a^	441	0.1 ± 0.5	17	0 ± 1	424	0.1 ± 0.5	0.37
Preoperative RV function^a^	434	0.1 ± 0.5	18	0.3 ± 0.9	416	0.1 ± 0.4	**0**.**04**
Ventricular physiology	510		21		489		—
Biventricular		405 (79.4)		19 (90.5)		386 (78.9)	
Single LV		49 (9.6)		0		49 (10.0)	
Single RV		56 (11.0)		2 (9.5)		54 (11.1)	
Diagnosis							
Aortic hypoplasia	510	26 (5.1)	21	1 (4.8)	489	25 (5.1)	1.00
Aortic stenosis	510	11 (2.2)	21	1 (4.8)	489	10 (2.0)	0.37
ASD	510	65 (12.7)	21	1 (4.8)	489	64 (13.1)	0.50
AVSD	510	100 (19.6)	21	4 (19.0)	489	96 (19.6)	1.00
Cardiomyopathy	510	6 (1.2)	21	0	489	6 (1.2)	1.00
Coarctation	510	28 (5.5)	21	2 (9.5)	489	26 (5.3)	0.32
Coronary anomaly	510	16 (3.1)	21	2 (9.5)	489	14 (2.9)	0.14
DORV	510	67 (13.1)	21	0	489	67 (13.7)	0.09
HLHS	510	38 (7.5)	21	4 (19.0)	489	34 (7.0)	0.06
Interrupted aortic arch	510	18 (3.5)	21	0	489	18 (3.7)	1.00
MAPCA	510	17 (3.3)	21	4 (19.0)	489	13 (2.7)	0.004
Pulmonary atresia	510	40 (7.8)	21	4 (19.0)	489	36 (7.4)	0.07
Pulmonary stenosis	510	26 (5.1)	21	0	489	26 (5.3)	0.62
Pulmonary vein stenosis	510	10 (2.0)	21	0	489	10 (2.0)	1.00
Severe mitral insufficiency	510	42 (8.2)	21	7 (33.3)	489	35 (7.2)	<0.001
Severe tricuspid insufficiency	510	47 (9.2)	21	4 (19.0)	489	43 (8.8)	0.12
TAC	510	14 (2.7)	21	0	489	14 (2.9)	1.00
TGA	510	93 (18.2)	21	3 (14.3)	489	90 (18.4)	0.78
Tricuspid atresia	510	23 (4.5)	21	0	489	23 (4.7)	0.62
VSD	510	120 (23.5)	21	7 (33.3)	489	113 (23.1)	0.30
Procedure							
Aortic arch repair	510	45 (8.8)	21	2 (9.5)	489	43 (8.8)	0.71
Aortic valve repair	510	15 (2.9)	21	1 (4.8)	489	14 (2.9)	0.47
Aortopulmonary shunt	510	22 (4.3)	21	6 (28.6)	489	16 (3.3)	**<0**.**001**
ASO	510	88 (17.3)	21	3 (14.3)	489	85 (17.4)	1.00
AVSD repair	510	85 (16.7)	21	4 (19.0)	489	81 (16.6)	0.76
BCPS	510	11 (2.2)	21	0	489	11 (2.2)	1.00
Coronary repair	510	13 (2.5)	21	3 (14.3)	489	10 (2.0)	**0**.**01**
DKS or Norwood procedure	510	39 (7.6)	21	2 (9.5)	489	37 (7.6)	0.67
DORV repair	510	34 (6.7)	21	0	489	34 (7.0)	0.39
Fontan procedure	510	59 (11.6)	21	0	489	59 (12.1)	0.15
Heart transplant	510	10 (2.0)	21	1 (4.8)	489	9 (1.8)	0.35
Pulmonary artery banding	510	9 (8.1)	21	1 (4.8)	489	8 (1.6)	0.32
Pulmonary atresia repair	510	16 (3.1)	21	0	489	16 (3.3)	1.00
Pulmonary vein repair	510	27 (5.3)	21	1 (4.8)	489	26 (5.3)	1.00
RV–PA conduit	510	51 (10.0)	21	3 (14.3)	489	48 (9.8)	0.46
TAC repair	510	16 (3.1)	21	1 (4.8)	489	15 (3.1)	0.49
Unifocalization	510	13 (2.5)	21	2 (9.5)	489	11 (2.2)	0.10
VSD closure		3 (14.3)					

ASD, atrial septal defect; ASO, arterial switch operation; AVSD, atrioventricular septal defect; BCPS, bilateral superior cavopulmonary shunt; DORV, double-outlet right ventricle; HLHS, hypoplastic left heart syndrome; MAPCA, major aortopulmonary collateral artery; RV–PA, right ventricle to pulmonary artery; TAC, truncus arteriosus communis; TGA, transposition of the great arteries; VSD, ventricular septal defect.

*n* represents the number of patients in the cohorts described in the columns to the right. The percentage is shown in parentheses.

Bold values denote statistical significance at the *p* < 0.05 level.

^a^ Ventricular function is numerically categorized as 0 = normal, 1 = mildly reduced, 2 = moderately reduced, 3 = severely reduced based on preoperative echocardiographic assessment.

### Settings of ECMO initiation

**Figure 1:**
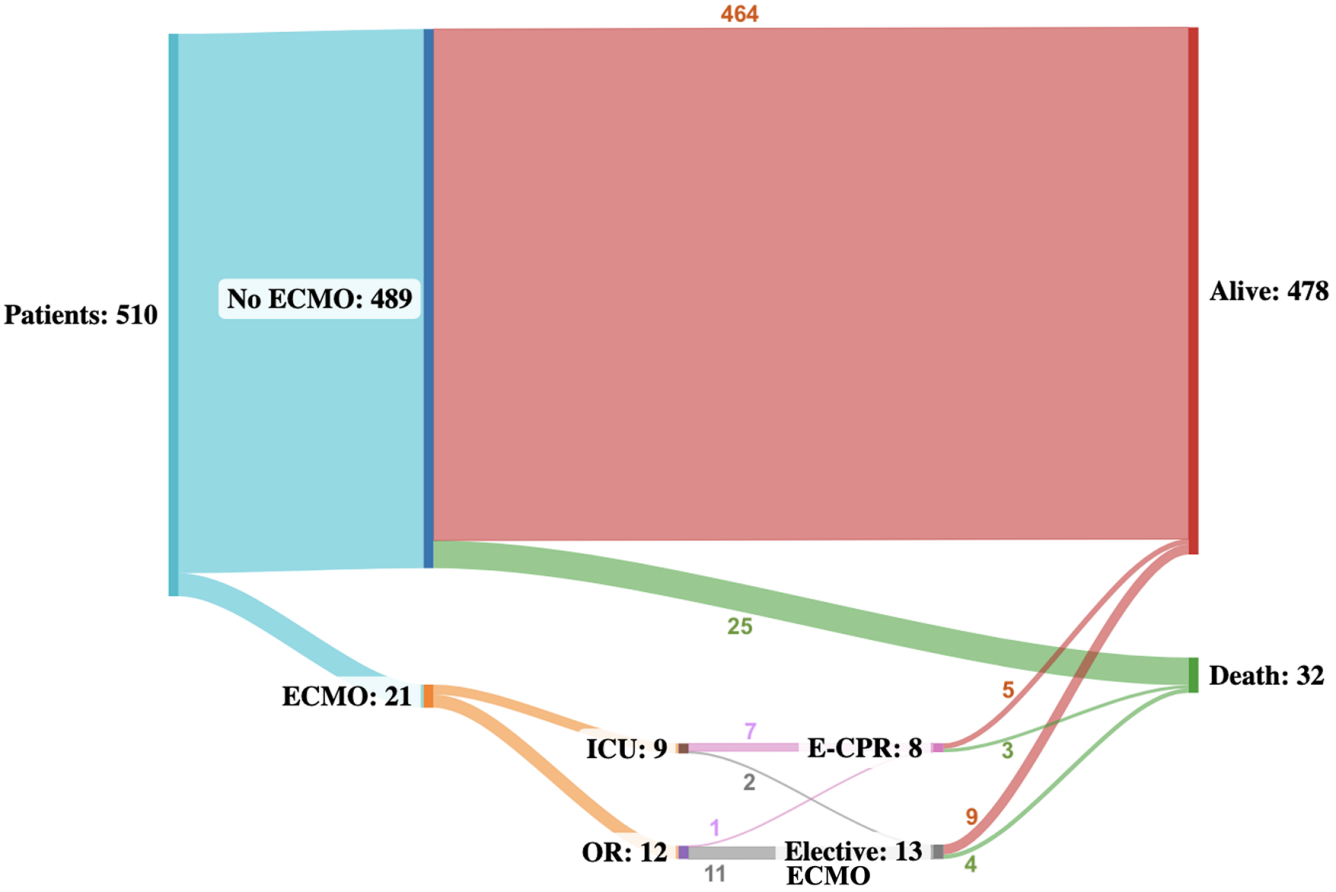
Sankey flowchart illustrating the event trajectory of all patients throughout a median follow-up of 10.8 (IQR: 2.6–22.6) months. Numbers describe the number of patients in each status. Alive and death are terminal states.

ECMO was more frequently initiated in the OR than in the ICU [12 (57%) vs. 9 (43%), Figure 1]. ECPR was more common with ICU cannulation than in the OR - 1 (8%) vs. 7 (78%)].

### Immediate postoperative clinical, echocardiographic, and biochemical outcomes

ECMO patients had a greater rate of postoperative severe mitral regurgitation (MR) [7/12 (33%) vs. 35/489 (7%), *p* < 0.001] and more severe LV dysfunction [6/21(29%) vs. 5/489 (1%), *p *< 0.001] with a similar rate of residual shunt (Table 2) on intraoperative transesophageal echocardiography, compared to non-ECMO patients. Intraoperatively postrepair, ECMO patients also possessed significantly higher lactate levels (7.1 ± 3.3 vs. 3.8 ± 2.4 mmol/L, *p *< 0.001) and VIS (27.6 ± 31.8 vs. 10.7 ± 9.9 μg/kg/min, *p *< 0.03) than those who did not receive ECMO.

**Table 2:** Postrepair echocardiographic, biochemical, and clinical outcomes.

Postoperative finding	*n*	Total	*n*	ECMO	*n*	No ECMO	*p*-value
Postoperative severe MR	510	42 (8.2)	21	7 (33.3)	489	35 (7.2)	**<0**.**001**
Residual ASD	510	112 (22)		5 (23.8)		107 (21.9)	0.79
Residual VSD	510	30 (5.9)		0		30 (6.1)	0.63
LV function	508		21		487		**<0**.**001**
Normal		419 (82.5)		11 (52.4)		408 (83.8)	
Mildly reduced		44 (8.7)		2 (9.5)		42 (8.6)	
Moderately reduced		34 (6.7)		2 (9.5)		32 (6.6)	
Severely reduced		11 (2.2)		6 (28.6)		5 (1.0)	
Intraoperative postrepair							
pH	462	7.32 ± 0.08	16	7.30 ± 0.11	446	7.32 ± 0.07	0.37
NIRS	489	61 ± 15	19	52 ± 19	470	61 ± 15	**0**.**045**
Lactate (mmol/L)	454	3.9 ± 2.5	16	7.1 ± 3.3	438	3.8 ± 2.4	**0**.**001**
SaO_2_ (%)	503	95 ± 11	19	85 ± 17	484	95 ± 11	**0**.**01**
VIS (μg/kg/min)			21	27.6 ± 31.8	489	10.7 ± 9.9	**0**.**03**
Death	510	32 (6.3)	21	7 (33.3)	489	25 (5.1)	**0**.**001**

ASD, atrial septal defect.

*n* represents the number of patients in the cohorts described in the columns to the right. The percentage is shown in parentheses.

Bold values denote statistical significance at the *p* < 0.05 level.

### Predictors of ECMO

Predictors of ECMO use in the OR were CPB duration, unplanned procedure, unifocalization, coronary repair, aortopulmonary shunt, residual severe MR, intraoperative lactate, and VIS immediately postrepair (Table 3). Predictors of ECMO use in the ICU were pulmonary arterioplasty, aortopulmonary shunt, residual severe tricuspid regurgitation (TR), and VIS on admission to the ICU.

**Table 3:** Univariate analysis of predictors of ECMO in the OR and in the ICU.

	Odds ratio for ECMO in the OR	*p*-value	HR for ECMO in the ICU	*p*-value
Age at the time of operation (years)	0.67 (0.32–1.41)	0.29	1.00 (1.00–1.00)	0.51
Prematurity	1.00 (0.20–4.88)	1.00	1.16 (0.23–5.75)	0.86
Planned procedure	0.16 (0.03–0.77)	**0**.**02**		
CPB duration (min)	1.03 (1.02–1.04)	**<0**.**001**	1.01 (1.00–1.02)	**0**.**03**
Cross-clamp duration (min)	1.01 (1.00–1.02)	0.07	1.01 (1.01–1.02)	**<0**.**001**
Diagnosis				
Aortopulmonary shunt	13.33 (3.67–48.40)	**<0.001**	7.91 (1.64–38.10)	**0**.**01**
Coronary repair	8.85 (1.73–45.26)	**0**.**01**	5.70 (0.71–45.54)	0.10
DKS or Norwood procedure	1.10 (0.14–8.75)	0.93	1.52 (0.19–12.13)	0.69
Pulmonary arterioplasty	1.40 (0.37–5.25)	0.62	5.29 (1.42–19.70)	**0**.**01**
Unifocalization	8.85 (1.73–45.26)	**0**.**01**		
Residual severe MR	8.90 (2.69–29.42)	**<0.001**	3.66 (0.76–17.62)	0.11
Residual severe TR	0.89 (0.11, 7.07)	0.92	5.07 (1.27, 20.25)	**0**.**02**
Intraoperative postrepair				
Lactate	1.52 (1.25–1.87)	**<0**.**001**	1.24 (1.00–1.55)	0.05
VIS	1.05 (1.03–1.08)	**<0**.**001**	1.04 (1.01–1.08)	**0**.**01**

The 95% confidence interval is shown in parentheses.

Bold values denote statistical significance at the *p* < 0.05 level.

### Survival

**Figure 2:**
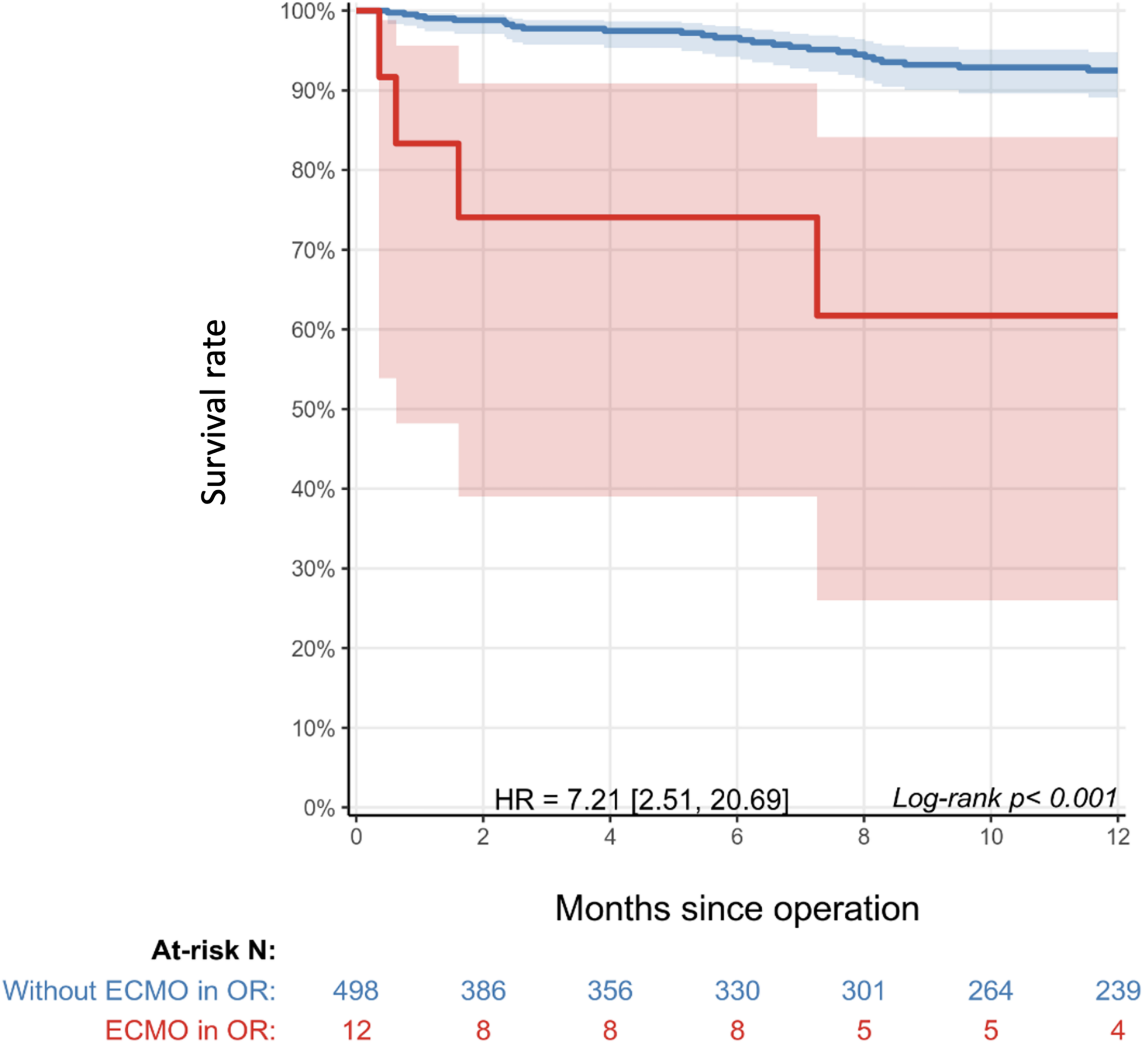
Kaplan–Meier survival curve of patients with ECMO initiation in the operating room (red) compared to those without ECMO initiation (blue). Shaded areas represent 95% confidence intervals. The table below the graph shows the number of patients at risk in each cohort over time. Administrative censoring was applied at year 1. HR: 7.2 (2.5–20.7), *p* < 0.001.

In-hospital survival was 90.5% and 98.0% in the ECMO and non-ECMO groups, respectively. At a median follow-up of 10.8 (IQR: 2.6–22.6) months, survival was 66.7% (*n* = 14/21) in the ECMO group and 94.9% (*n* = 464/489) in the non-ECMO group. Of patients who received ECMO, survival at 2 and 10 years was 75.0% and 62.5% compared to 98.7% and 93.6% among patients who did not receive ECMO in the OR, respectively (Figure 2). Of the seven deaths in the ECMO group, four occurred after elective ECMO initiation intraoperatively and three after ECPR in the ICU. Of the 32 total deaths, 12 occurred in the ICU, 3 in the non-ICU setting within the hospital, and 17 in the community. The baseline characteristics of ECMO patient survivors and non-survivors are shown in Supplementary Table S1.

### Predictors of mortality

Univariate analysis revealed that the predictors for mortality in the general cohort were prematurity, Damus–Kaye–Stansel or Norwood procedure, pulmonary vein repair, coronary repair, moderate-to-severe LV dysfunction, residual severe TR, and intraoperative lactate (Table 4). The HR for mortality related to ECPR was 15.42 compared to 5.26 for elective ECMO, but the difference did not reach statistical significance (*p* = 0.162). Patients who received ECMO in the OR had significantly higher mortality than those without ECMO in the OR [HR: 7.21 (2.51–20.69), *p* < 0.001], with the greatest risk of death seen in the first 2 months postrepair.

**Table 4:** Univariate analysis of predictors of mortality.

	HR for mortality	*p*-value
Age at the time of operation (years)	1.00 (1.00–1.00)	0.05
Prematurity	2.61 (1.25–5.47)	**0**.**01**
Planned procedure	0.53 (0.13–2.22)	0.39
CPB duration (min)	1.01 (1.0–1.01)	**0**.**01**
Cross-clamp duration (min)	1.00 (1.00–1.01)	**0**.**04**
Procedure		
Aortopulmonary shunt	2.80 (0.98–7.98)	0.05
Coronary repair	3.63 (1.11–11.93)	**0**.**03**
DKS or Norwood	4.29 (1.99–9.28)	**<0**.**001**
Pulmonary arterioplasty	1.54 (0.71–3.34)	0.27
Pulmonary vein repair	3.72 (1.30–10.65)	**0**.**03**
Postrepair echocardiography		
Moderate-severe LV dysfunction	5.10 (1.50–17.32)	**0**.**01**
Residual severe MR	1.27 (0.39–4.18)	0.69
Residual severe TR	6.06 (2.86–12.83)	**<0**.**001**
Intraoperative postrepair		
Lactate^a^	1.36 (1.22–1.50)	**<0**.**001**
VIS^a^	1.03 (1.02–1.04)	**<0**.**001**
ICU admission postrepair		
Lactate^b^	1.34 (1.19–1.50)	**<0**.**001**
VIS^b^	1.05 (1.03–1.07)	**<0**.**001**
LCOS	2.18 (0.76–6.22)	0.15
ECMO type		
ECPR	15.42 (5.27–45.12)	**<0**.**001**
Elective ECMO	5.26 (1.58–17.53)	**0**.**01**
ECMO in the OR	7.21 (2.51–20.69)	**<0**.**001**
ECMO in the ICU	9.28 (2.77–31.1)	**<0**.**001**

The 95% confidence interval is shown in parentheses.

Bold values denote statistical significance at the *p* < 0.05 level.

^a^ Measurements taken at different time points are annotated by superscript numbers: in the operating room after surgical repair.

^b^ Postoperative ICU admission.

## Discussion

### Prevalence of postcardiotomy ECMO

This single-center retrospective cohort study analyzed the predictors, settings, and outcomes of ECMO initiation among 510 consecutive pediatric patients who underwent moderate-to-high-risk cardiac surgery; among them, 21 (4.1%) received postoperative ECMO, of which there were 10 cases of ECPR. In the contemporary era, the rate of postcardiotomy ECMO in pediatric patients and neonates with congenital heart disease ranges from 3% to 4% (11–13), with a rate of 2.5% reported in systematic analysis and meta-analysis of 26 studies by Cho et al. (14). ECPR comprises 27%–39% of total ECMO cases (6, 11, 13), with ECPR rates as high as 50% described in a cohort of patients aged ≤6 weeks (12). Our observed ECMO and ECPR rates were within the upper range of the existing literature due to the inclusion criteria of RACHS-1 ≥ 3.

### Biochemical predictors of ECMO initiation

In keeping with findings from other centers, arterial lactate and VIS were identified as predictors of postoperative ECMO after pediatric cardiac surgery (12). In the ECMO group, the intraoperative median VIS and arterial lactate were 16.6 (8.6–35) μg/kg/min and 7.7 (4.5–10) mmol/L, respectively. The multivariate regression analysis by Kuraim et al. identified peak VIS >30 (OR: 1.02; *p *< 0.001) and peak lactate >6 mmol/L (OR: 1.2; *p *= 0.003) as makers for ECMO initiation within 48 h postrepair (12). Conversely, Charpie et al. proposed that up-trending serial lactate levels were a more sensitive and specific indicator for the composite outcome of ECMO or early death than singular lactate levels. They found that initial lactate >6 mmol/L and increasing lactate of >0.75 mmol/L/h had positive predictive values of 38% and 100%, respectively (15). Although lactate was not trended in our study, the poor prognostication associated with lactate >6 mmol/L, often referred to in the literature, is also reflected in the findings. The median VIS score in our ECMO group was comparatively lower, possibly owing to a lower threshold for ECMO initiation before maximally tolerated vaso-inotropic agents are employed, as suggested by 9 out of 21 patients being cannulated in the OR.

### Surgical predictors of ECMO initiation

Our univariate analysis identified aortopulmonary shunt, unifocalization, and coronary repair as predictors of intraoperative ECMO. Aortopulmonary shunts were often performed in conjunction with other procedures, including unifocalization, truncus arteriosus repair, pulmonary vein repair, and pulmonary arterioplasty. Aside from shunt thrombosis, the indication for ECMO in the aortopulmonary cohort can be driven by inadequate repair of other cardiac lesions. Unifocalization being a risk factor for intraoperative ECMO could be attributed to the sequelae of pulmonary edema and right ventricular (RV) dysfunction associated with major aortopulmonary collateral arteries or shunt run-off causing coronary hypoperfusion in cases of staged repair. Suboptimal coronary repair or collateral coronary injury during repair of the primary lesion poses difficulty with weaning from CPB, hence greater odds of intraoperative ECMO initiation.

Although there is a significant overlap between predictors of ECMO in the OR and in the ICU, pulmonary arterioplasty and residual severe TR were recognized as predictors of ECMO institution in the ICU, but not in OR. The severity of right heart lesions and their associated RV dysfunction are often unmasked in the ICU after perioperative hypotension and hypovolemia are treated. The association of ICU ECMO with pulmonary arterioplasty was initially hypothesized to be due to baseline RV dysfunction, but all three patients had normal functioning RV. However, residual pulmonary artery stenosis was noted in two of the three patients, with one of the patients also demonstrating additional preoperative moderate LV dysfunction.

In a meta-analysis of 16 heterogeneous cases of pediatric ECMO for CHD by Wu et al., the pooled estimate of residual lesions requiring reoperations was 14.9% (*I*^2^ = 80.3%) (16). This percentage was lower than in a large observational study by Agarwal et al., which found hemodynamically significant residual lesions requiring reintervention in 35 (28%) of 119 postoperative pediatric cardiac surgery patients, with the predominant lesion being branch pulmonary artery stenosis (*n* = 7/13) (17). They reported a superior rate of decannulation (*p *= 0.004) and in-hospital survival (*p *= 0.025) when residual lesions were identified in the first 3 days of ECMO support (17). Our findings corroborated the importance of avoiding residual defects, particularly residual pulmonary artery stenosis, with certain lesions being harder to appreciate until hemodynamic optimization in the ICU.

### ECMO survival and predictors of mortality

The in-hospital survival of postcardiotomy pediatric ECMO in this study was 90.5%. Historically, it was reported to be 37% at our institution (18), which is similar to the rates reported by other single-center studies of 37%–41% (17, 19). In a multicenter study involving 998 children from 37 American centers, Gupta et al. reported postoperative ECMO in-hospital survival of 48.1% (20). A meta-analysis of 43 international studies showed a pooled in-hospital mortality estimate of 56.8% (95% CI, 52.5%–61.0%, *I*^2^ = 74.2%) after pediatric CHD surgery (16). In that same study, multivariate meta-regression revealed single-ventricle physiology and renal failure as independent risk factors for in-hospital non-survival (16). The results from our study demonstrated comparably better post-ECMO survival than current literature; however, the mortality of surgery in single-ventricle physiology remains notably high and accounts for four of the seven deaths in our ECMO group.

Lactate is a well-investigated marker of mortality in the ECMO population and has been used to inform appropriateness for withdrawal of care (21). Our study revealed intraoperative and end-of-surgery lactate and VIS to be predictors of mortality. Similarly, Shah *et al*. also described high arterial lactate at the onset of ECMO to be a predictor of non-survival (lactate 14.4 ± 7.5 mmol/L among non-survivors, *p *= 0.004). Merkle-Storms et al. found the lactate trend at 3–12 h after starting ECMO to be indicative of prognosis, while pre-ECMO and peak lactate levels were similar between survivors and non-survivors (21). Conversely*,* Baslaim et al. demonstrated no significant difference in serum lactate within 72 h of ECMO initiation between those who survived to hospital discharge and those who did not (22). Prognostication is a multifaceted and dynamic process with case-by-case nuances. Whether trended or stand-alone, the lactate level is one of many markers to inform the larger clinical picture.

### Location of ECMO initiation

We found similar survival rates for ECMO cannulation in the OR and ECMO cannulation in the ICU. This is consistent with historical in-hospital survival data from our institutions (40% in OR vs. 37% in the ICU or the cath lab; *p* = 0.62) (6). Many pediatric studies that have explored the setting of ECMO initiation in the OR vs. ICU also reported no statistically significant difference in survival between locations (3, 11, 23, 24). Only one study demonstrated significantly better in-hospital survival when ECMO runs were initiated in the OR rather than in the ICU (64% vs. 29%, *p *= 0.003), partially because 56% of their ICU ECMO cohort were ECPR cases (25). Although not specific to postcardiotomy, a recent multicenter pediatric registry study of ECPR for in-hospital arrest showed an adjusted mortality odds ratio of 1.04 (95% CI, 1.01–1.07) for each 5 additional minutes between CPR and the start of ECMO (26). Our results are in line with the contemporary understanding that hemodynamic status before ECMO portends survival, rather than the environment of cannulation.

### ECPR vs. ECMO

There was no statistically significant survival difference between ECPR and elective ECMO. Our previous institutional data also demonstrated no survival disadvantage of ECPR (*p* = 0.18); in fact, survival was 46% and 35% in ECPR and ECMO groups, respectively (6). Shah et al. substantiated that postoperative CPR in the ICU at the time of ECMO initiation did not result in poorer survival (*p* = 0.59) in the CHD population (19). The 2023 Extracorporeal Life Support Organization registry-based retrospective cohort study of 2,155 CHD neonatal ECMO cases continued to show no survival difference in ECPR vs. ECMO (*p* = 0.31) (27).

Given the relative infrequency of ECPR, two meta-analyses on postcardiotomy pediatric ECMO attempted to answer whether ECPR carries worse in-hospital survival through subgroup analyses. The results were mixed. Pooled ECPR survival in a subgroup analysis involving 12 studies was 0.37% (95% CI, 0.29%–0.46%, *I*^2^ = 32%, *p* = 0.13), with the authors concluding lower mortality in ECMO for LCOS than ECPR (14). Pooled in-hospital mortality rates of 18 studies with ≤50% incidence of ECPR and 10 studies with >50% incidence of ECPR were 58.1% and 58.9%, respectively (16). In our study, ECPR had a threefold higher mortality hazard ratio than elective ECMO but failed to reach statistical significance. Early anticipation of ECMO requirement is needed to prevent ECPR and is widely adopted despite the lack of consensus or definitive survival benefit of elective or end-of-case ECMO.

## Limitations

Data were retrospectively collected and derived from a single center. The study is inherently limited in generalizability by its retrospective nature and modest sample size, which precluded competing risk analysis. Although a standardized protocol for the management of all patients on ECMO was followed, this underwent some evolution over the 9 years after data collection was completed. Multiple intensivists were involved in the care of the patients with expectable variations in practice.

## Conclusion

In pediatric patients undergoing CHD repair of moderate-to-severe complexity, ECMO can be initiated for the inability to wean from CPB or as rescue therapy for patients in hemodynamic extremis in the early postoperative setting. For patients who require ECMO, mortality remains considerable. CPB time, moderate-to-high LV dysfunction, significant residual lesions, elevated lactate, and VIS emerged as predictors of ECMO initiation. Risk factors for mortality include similar predictors to those for ECMO, with ECMO itself being a predictor of death and ECPR being particularly high-risk. Given the accentuated mortality of ECPR events in the ICU, prophylactic and early ECMO initiation in patients with multiple risk factors could be a promising strategy for further investigation.

## Data Availability

The original contributions presented in the study are included in the article/Supplementary Material; further inquiries can be directed to the corresponding author.
