# Supplementary material for: Early outcomes of moderate-to-high-risk pediatric congenital cardiac surgery and predictors of extracorporeal circulatory life support requirement
Source: Front Pediatr. 2024 Mar 8;12:1282275. doi: 10.3389/fped.2024.1282275 (PMC10957634; doi:10.3389/fped.2024.1282275)
Supplement: Supplementary file 1 [file Table1.docx]

Supplementary Material

# Supplementary Figures and Tables

**Table S1.** Baseline and operative characteristics of ECMO patients

|  | n | Survival | n | Non-survival | *p*-value |
| --- | --- | --- | --- | --- | --- |
| Age at time of operation (d) | 14 | 260 ± 256 | 7 | 57 ± 90 | 0.016 |
| Prematurity | 10 | 0 (0) | 7 | 4 (57) | 0.015 |
| Procedure planned | 14 | 14 (100) | 7 | 5 (71) | 0.100 |
| CPB duration (min) | 13 | 263 ± 98 | 7 | 226 ± 105 | 0.46 |
| Cross-clamp duration (min) | 13 | 156 ± 97 | 7 | 110 ± 74 | 0.26 |
| Preoperative LV function | 12 |  | 5 |  | 0.51 |
| Normal |  | 11 (92) |  | 4 (80) |  |
| Moderately reduced |  | 0 (0) |  | 1 (20) |  |
| Severely reduced |  | 1 (8) |  | 0 (0) |  |
| Preoperative RV function | 12 |  | 6 |  | 0.73 |
| Normal |  | 10 (83) |  | 5 (83) |  |
| Mildly reduced |  | 0 (0) |  | 1 (17) |  |
| Moderately reduced |  | 1 (8) |  | 0 (0) |  |
| Severely reduced |  | 1 (8) |  | 0 (0) |  |
| Ventricular physiology | 14 |  | 7 |  | - |
| Biventricular |  | 14 (100) |  | 5 (71) |  |
| Single LV |  | 0 |  | 0 |  |
| Single RV |  | 0 |  | 2 (29) |  |
| Diagnosis |  |  |  |  |  |
| Aortic hypoplasia | 14 | 1 (7) | 7 | 0 (0) | 1.00 |
| Aortic stenosis | 14 | 1 (7) | 7 | 0 (0) | 1.00 |
| ASD | 14 | 1 (7) | 7 | 0 (0) | 1.00 |
| AVSD | 14 | 4 (29) | 7 | 0 (0) | 0.25 |
| Coarctation | 14 | 1 (7) | 7 | 1 (14) | 1.00 |
| Coronary anomaly | 14 | 1 (7) | 7 | 1 (14) | 1.00 |
| HLHS | 14 | 1 (7) | 7 | 3 (43) | 0.088 |
| MAPCA | 14 | 3 (21) | 7 | 1 (14) | 1.00 |
| Mitral valve dysfunction | 14 | 1 (7) | 7 | 0 (0) | 1.00 |
| Pulmonary atresia | 14 | 2 (14) | 7 | 2 (29) | 0.57 |
| TGA | 14 | 3 (21) | 7 | 0 (0) | 0.52 |
| VSD | 14 | 4 (29) | 7 | 3 (43) | 0.64 |
| Procedure |  |  |  |  |  |
| Aortic arch repair | 14 | 1 (7) | 7 | 1 (14) | 1.00 |
| Aortic valve repair | 14 | 1 (7) | 7 | 0 (0) | 1.00 |
| Aortopulmonary shunt | 14 | 3 (21) | 7 | 3 (43) | 0.35 |
| ASD closure | 14 | 2 (14) | 7 | 0 (0) | 0.53 |
| ASO | 14 | 3 (21) | 7 | 0 (0) | 0.52 |
| Atrial septectomy | 14 | 0 (0) | 7 | 1 (14) | 0.33 |
| Atrioventricular valve repair | 14 | 2 (14) | 7 | 1 (14) | 1.00 |
| AVSD repair | 14 | 4 (29) | 7 | 0 (0) | 0.25 |
| Coarctation repair | 14 | 1 (7) | 7 | 0 (0) | 1.00 |
| Coronary repair | 14 | 1 (7) | 7 | 2 (29) | 0.25 |
| DKS or Norwood procedure | 14 | 0 (0) | 7 | 2 (29) | 0.100 |
| Heart transplant | 14 | 1 (7) | 7 | 0 (0) | 1.00 |
| Pulmonary arterioplasty | 14 | 3 (21) | 7 | 5 (71) | 0.056 |
| Pulmonary artery banding | 14 | 1 (7) | 7 | 0 (0) | 1.00 |
| Pulmonary vein repair | 14 | 0 (0) | 7 | 1 (14) | 0.33 |
| RV-PA conduit | 14 | 3 (21) | 7 | 0 (0) | 0.52 |
| TAC repair | 14 | 1 (7) | 7 | 0 (0) | 1.00 |
| Unifocalization | 14 | 2 (14) | 7 | 0 (0) | 0.53 |
| VSD closure | 14 | 2 (14) | 7 | 1 (14) | 1.00 |

N represents the number of patients in the cohorts described in the columns to the right. Percentage shown in brackets. *Ventricular function is numerically categorized as 0 = normal, 1 = mildly reduced, 2 = moderately reduced, 3 = severely reduced based on pre-operative echocardiographic assessment. ASD, atrial septal defect; ASO, arterial switch operation; AVSD, atrioventricular septal defect; CPB, cardiopulmonary bypass; DKS, Damus-Kaye-Stansel procedure; ECMO, extracorporeal membrane oxygenation; HLHS, hypoplastic left heart syndrome; LV, left ventricle; MAPCA, major aortopulmonary collateral artery; RV, right ventricle; RV-PA, right ventricle to pulmonary artery; TAC, truncus arteriosus communis; TGA, transposition of the great arteries; VSD, ventricular septal defect.
